# World Trade Center Disaster Exposure-Related Probable Posttraumatic Stress Disorder among Responders and Civilians: A Meta-Analysis

**DOI:** 10.1371/journal.pone.0101491

**Published:** 2014-07-21

**Authors:** Bian Liu, Lukman H. Tarigan, Evelyn J. Bromet, Hyun Kim

**Affiliations:** 1 Department of Population Health, Hofstra North Shore-LIJ School of Medicine, Great Neck, New York, United States of America; 2 Department of Work Environment, University of Massachusetts Lowell, Lowell, Massachusetts, United States of America; 3 Department of Psychiatry and Behavioral Science, State University of New York-Stony Brook, Stony Brook, New York, United States of America; University of Pennsylvania, United States of America

## Abstract

The World Trade Center (WTC) disaster on September 11, 2001 was an unprecedented traumatic event with long-lasting health consequences among the affected populations in the New York metropolitan area. This meta-analysis aimed to estimate the risk of probable posttraumatic stress disorder (PTSD) associated with specific types of WTC exposures. Meta-analytical findings from 10 studies of 3,271 to 20,294 participants yielded 37 relevant associations. The pooled summary odds ratio (OR) was 2.05 (95% confidence interval (CI): 1.82, 2.32), with substantial heterogeneity linked to exposure classification, cohort type, data source, PTSD assessment instrument/criteria, and lapse time since 9/11. In general, responders (e.g. police, firefighters, rescue/recovery workers and volunteers) had a lower probable PTSD risk (OR = 1.61; 95% CI: 1.39, 1.87) compared to civilians (e.g. residents, office workers, and passersby; OR = 2.71, 95% CI: 2.35, 3.12). The differences in ORs between responders and civilians were larger for physical compared to psychosocial exposure types. We also found that injury, lost someone, and witnessed horror were the three (out of six) most pernicious exposures. These findings suggest that these three exposures should be a particular focus in psychological evaluation and treatment programs in WTC intervention and future emergency preparedness efforts.

## Introduction

The World Trade Center (WTC) disaster on September 11, 2001 (9/11) was an unprecedented traumatic event to the responders and civilians in the New York metropolitan area and beyond. In the wake of the disaster, many programs were established to provide physical and mental health screening, monitoring and/or treatment service to affected individuals. Three major programs established in the New York metropolitan area are: the Fire Department of the City of New York Medical Monitoring Program (the FDNY), the WTC Health Registry (the Registry), and the WTC Health Program (WTC-HP, formally known as the WTC Medical Monitoring and Treatment Program or MMTP). The FDNY program was implemented in 2001 to screen and monitor FDNY members involved in the rescue and recovery efforts of 9/11. This group consists predominantly of active firefighters, but also emergency medical service workers, FDNY administrative personnel, and some retired FDNY as well. The Registry was established in July 2002 by the New York City Department of Health and Mental Hygiene in collaboration with the Agency for Toxic Substances and Disease Registry. Registry enrollees include rescue/recovery workers and volunteers (i.e. non-traditional responders), passersby, and school children and staff, residents and office workers in lower Manhattan. The WTC-HP is a Center for Disease Control and Preventions (CDC) funded consortium of 5 centers including the Department of Community and Preventive Medicine at the Mount Sinai School of Medicine, the Bellevue/New York University Occupational and Environmental Medicine Clinic, the State University of New York-Stony Brook, the Center for the Biology of Natural Systems at Queens College, and the Clinical Center of the Environmental & Occupational Health Sciences Institute at the University of Medicine and Dentistry of New Jersey-Robert Wood Johnson Medical School in New Jersey. Founded in July 2002, the program monitors and treats police and non-traditional responders (e.g. recovery/rescue workers and volunteers, construction workers, transportation workers, etc.) who participated in the rescue/clean-up/recovery work from 9/11/2001 until 12/31/2001.

During more than a decade after the 9/11 event, a number of studies from the three centers [1]–[13] and from other WTC research programs [14],[15] investigated a range of physical and mental health conditions among both responders and civilians. Among the mental health conditions, probable posttraumatic stress disorder (PTSD), measured with check-lists tailored to the event, stands out as one of the major syndromes that appears to have endured over the decade following the disaster [16],[17]. Recent reviews [17]–[21] also point to specific risk factors that were found to be associated with probable PTSD in these samples, including sociodemographic and occupational characteristics, types of exposure, social support, and medical comorbidity. The focus of this study is to extend the scope of these reviews by providing a quantitative effect size estimate of PTSD risks attributable to specific WTC exposures, while taking into account the discrepancies in study designs.

By studying research results accumulated more than a decade since the event, we aim to understand the psychological health impact of 9/11 in an effort to provide insights that could enhance current intervention and future disaster preparedness programs. To this end, we conducted a meta-analysis to quantify the odds ratio (OR) for probable PTSD associated with specific WTC exposures, and to examine whether discrepancies in aspects of study design such as WTC exposure classification and cohort type (i.e. responders vs. civilians) affected PTSD risk.

## Methods

### Data source and searches

Relevant studies were identified by searching PubMed databases for all published articles up to 22 April 2013, using the relevant search terms such as “world trade center”, “WTC”, “world trade center disaster”, “WTCD”, “September 11”, “9/11”, “posttraumatic stress disorder”, “PTSD”, “post traumatic stress disorder”, “world trade center medical monitoring and treatment program”, “WTC-MMTP”, “Medical Monitoring and Treatment Program”, “MMTP”, “world trade center health registry”, “WTC-HR”, “WTCHR”, “Health Registry”, “HR”, “New York City Department of Health and Mental Hygiene”, “NYC DOHMH”, “NYC”, “DOHMH”, “Fire Department of the City of New York”, “FDNY”, “medical monitoring program”, “MMP”, “FDNY-WTC-MMP”, “WTC-MMP”, and “FDNY-MMP”.

### Inclusion/Exclusion criteria

We selected studies for the meta-analysis based on the following criteria: 1) published in an English-language journal; 2) peer-reviewed; 3) original research papers; 4) focused on adult populations; 5) conducted in the New York metropolitan area; 6) specified the PTSD measurement instrument and criteria used; 7) specified exposure levels; 8) listed specific numbers of study participants who were classified with and without PTSD corresponding to the exposure levels.

### Data extraction

Data relevant to the associations between WTC exposure and PTSD risks were extracted. Eligible articles and extracted data were examined by three investigators (B.L., L.H.T., and H.K.). Data extracted included cohort types (e.g. firefighters, police, non-traditional responders, residents, office workers, and passersby), data source (i.e. FDNY, Registry, WTC Health Program, and others), exposure types, PTSD assessment instrument/criteria, sample size, probable PTSD prevalence, the number of subjects with and without probable PTSD (P+/P-) among those with high vs low or no WTC exposure.

### Statistical analysis

We used the DerSimonina-Laird (DL) random-effects model [22] to calculate the summary effect size of OR and the 95% confidence interval (CI) (i.e. OR [95% CI]). Analysis was conducted using R software with the *metfor* Package [23]. Variability across individual ORs was assessed by five variables: the between-study variance (τ^2^); the standard error (SE) of the overall population effect size estimate; the Cochran's Q-test (p-value reported here); I^2^ value; and H^2^ statistics [24]. For each study, we approximated an average lapse time based on the differences between 9/11 and the earliest and latest enrollment time (e.g. if a study enrollment period was 2001–2005, the average lapse time was (0+4)/2 = 2 years). We explored the influence of four potential moderators, namely, cohort type, WTC program, PTSD measure, and lapse time. To do this, we used mixed-effects models and included one moderator in the model at a time.

Sensitivity analysis was conducted to assess potential substantial changes in the summary effect size by a few individual data points. This was done by using the *influence()* function, which provides visual examination, and by the *leav1out()* function, which is conducted by repeatedly fitting the DL model (without moderators) while leaving out one study at a time. We also visually examined the symmetry in the funnel plot for publication bias. In addition, asymmetry of the funnel plot was assessed using the rank correlation analysis (i.e. the Begg's method, [25]) and linear regression analysis (i.e. the Egger's method,[26]). Both the Begg's and Egger's tests were used to examine if there were significant correlations between the effect estimates and their variances.

## Results

### Systematic search results

**Figure 1:**
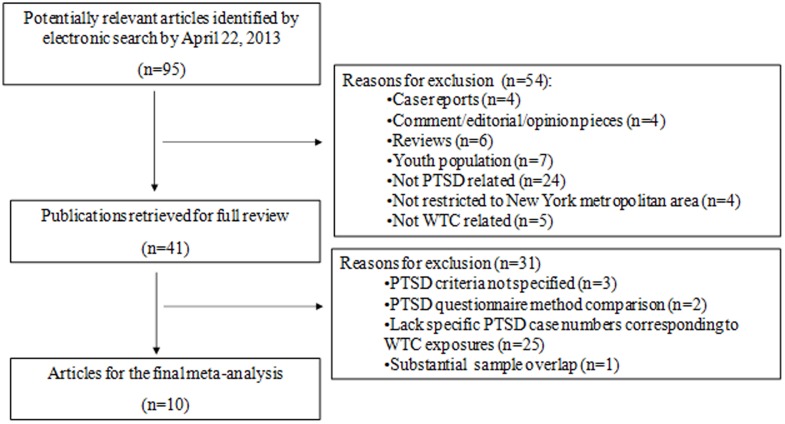
Flow chart of study selection.

Of the 95 English-language articles resulting from the search, 54 studies were excluded after reviewing the abstracts in the first round of screening (Figure. 1) due to at least one of the following reasons: case report (n = 4), comment/editorial/opinion piece (n = 4), review paper (n = 6), youth population (n = 7), not PTSD related (n = 24), not restricted to the New York metropolitan area (n = 4), and not WTC related (n = 5). An additional 31 studies were excluded after reviewing the full-text due to at least one of the following reasons: PTSD criteria not specified (n = 3), papers focused on validating a modified PTSD questionnaire (n = 2), lack of specific numbers of study participants who were classified with and without PTSD that corresponded to the exposure levels presented in the paper (n = 25), and significant sample overlap with another paper included in the analysis (n = 1). Our search strategy and inclusion/exclusion criteria resulted in a total of 10 articles for the current meta-analysis ([1]–[7] [9] [12] [13]; Table 1). Four papers (b–d, and f) were from the Registry, three studies (a, h, and j) were from the FDNY, and three from the WTC-HP (e, g, and i).

**Table 1:** Descriptions of the ten WTC studies included in the meta-analysis.

ID	Articles	Cohort Types	WTC Programs	Enrollment period	PTSD assessment (instrument/criteria)	PTSD (%)	Total (n)	Sample Characteristics ^d^
a	Berninger et al., 2010	firefighters	FDNY	2001–2005	Modified PCL ^a^	14.4	10074	Male (100); Age (39.6±7.5); White (93.8)
b	Brackbill et al., 2009	non-traditional responders	Registry	2003–2004	PCL (≥44)	22.9	20294	Male (62);
		residents		&		21.3	5852	Age (25–44);
		office workers		2006–2007		25.2	14718	White (70.8)
		passersby				29.2	2087	
c	DiGrande et al., 2008	residents	Registry	2003–2004	PCL (≥44 & DMS-IV)	12.6	11037	Male (44.6); Age (46);White (62.1)
d	DiGrande et al., 2011	office workers	Registry	2003–2004	PCL (≥50)	15	3271	Male (58.8); Age (40.8±10.9); White (68.2)
e	Luft et al., 2012	Police	WTC Health Program	2002–2008	PCL (≥50)	5.9	8508	Male (85); Age (40.8±6.6)
		non-traditional responders				23	12333	Male (86.1); Age (44.4±9.9)
f	Nair et al., 2012	residents	Registry	2003–2004	PCL(≥44 &DSM-IV)	8.5^b1^	16363	Male (48.3); Age (18–65+)
				& 2006–2007		5.8^b2^	16363	Male (48.3);White (67.3); Age (25–44)
g	Pietrzak et al., 2012	police	WTC Health Program	2002–2008	PCL (≥50 & DSM-IV)	5.4^c^	8466	Male (85.3); Age (35–59); White (48.9)
h	Soo et al., 2011	firefighters	FDNY	2006–2007^d^	PCL (≥44 & DSM-IV)	7.7	4343	Male (100); Age (38.9±7.9); White (93.7)
i	Stellman et al., 2008	police	WTC Health Program	2002–2006	PCL (≥50)	11.1	10132	Male (87.3); Age (median:40); White (64)
j	Webber et al., 2011	Firefighters & EMS workers	FDNY	2007–2010	PCL (≥44 & DSM-IV)	6.9	10867	Male(89.2); Age (25–44); White (89.2)

**Note**: PTSD (%)  =  probable PTSD prevalence. n =  total numbers of participants. FDNY  = Fire Department of the City of New York. PCL = PTSD Checklist-Civilian Version, DSM-IV = Diagnostic and Statistical Manual of Mental Disorders, 4^th^ edition. ^a^, PTSD assessed by a modified PCL. ^b1^, those with PTSD alone; ^b2^, those with both PTSD and lower respiratory symptoms. ^c^, full PTSD. ^d^, the percentages (%) of male sex and white ethnic/race, and age in years (age ± standard deviation, median, or age bracket with the largest percentage) were shown in (). ^d^, results from 2006–2007 was used in this meta-analysis.

### Sample Characteristics

The 10 articles we identified had cohorts that ranged from N =  3,271 to N = 20,294 (Table 1). Among them, five studies (a, e, g, h, i, and j) focused solely on responders, three papers (c, d, and f) focused solely on civilians, and one article (b) included a mix of responders and civilians. The participants were enrolled at different time relative to 9/11 ranging from a few months to 9 years, with an estimated average lapse time of 3.5 years. The responders were predominantly male (>85%) and white, ranging from 49–64% of police and up to 94% of firefighters. The male-to-female ratios were more balanced among civilians, and 62–71% of them were white.

Overall the pooled samples included three cohorts from the three major WTC program centers that captured the diverse populations affected by the WTC disaster in the New York Metropolitan area. The overlaps in participants among the three programs ranged from less than 1% between the WTC-HP and FDNY to approximately 20% for responders between the Registry and WTC-HP [27]. The total number of FDNY participants ranged from 1159 to 8869 for each of the nine survey cycles between 2001 and 2010 [6]. One longitudinal study of FDNY ([28]; not included in this meta-analysis) reported an approximately 83% of the baseline enrollees participated in the 3–4 years follow-up. For the Registry, approximately 68% of the participants from the Wave 1 enrollment (2003-2004) also participated in Wave 2 (2006–2007) [2]. Within the WTC HP during the period of 2002–2012, the average number of participation of follow-up examinations was 1.1.

To minimize the shared sample problem while capture the maximum overall sample size, cohort types, and exposure categories for the meta-analysis, we carefully selected these 10 studies. The final extracted data used for the meta-analysis were presented in Table S1 as supplement information. In general, individual data with the largest number of participants were used during the analysis. For example, for civilian residents with exposure of “witnessed horror” and “injury”, data from DiGrande et al (n = 11037; [4]) were used instead of those from the study by Brackbill et al. (n = 5852; [2]). Further, analysis was conducted on a subset of data (Table S1) with minimal shared samples within the same exposure type.

### Assessment of WTC exposure

A total of six types of WTC exposures was summarized from the 10 studies with original and derived exposure types presented in Table 2. Overall, these exposures were coarsely grouped into two major categories: those that focused on physical exposure (i.e. arrival time, dust cloud, injury, and work duration) and those focused on psychosocial aspects (i.e. lost someone and witnessed horror). While there were more exposure types listed in the original articles (32 distinct exposure types; data not shown), many of them were unique to a single study, and thus were not applicable to the goals of the meta-analysis. That is, each of the 6 exposure types in this review (Table 2) had at least two individual OR estimates.

**Table 2:** Summary of the six WTC exposure types from the ten studies included in the meta-analysis.

Exposure types		Studies	Exposure classifications	
(used in the Meta-Analysis)			Summarized	Original
physical	Arrival Time	a , h, j	9/11-9/12	level 1: am on 9/11;
exposure	(Early vs		(sum of levels 1–3)	level 2: pm on 9/11;
	Otherwise*)		vs level 4*	level 3: day 2;
				level 4: day 3–14 *
		b	9/11	level 1: 9/11 (on pile);
			(sum of levels 1–2)	level 2: 9/11 (other WTC site);
			vs	level 3: 9/12-9/17 (any WTC site);
			otherwise (sum of levels 3–4)	level 4: 9/18/2001-6/2002, any WTC site *
		g	9/11 or 9/12	vs otherwise *
		i	Present 9/11-9/12:	Yes vs No*
	Dust Cloud	b, f	Yes (sum of levels 1–2) vs None*	level 1: intense; level 2: some; level 3: none*
	(Yes vs No*)	c, d	Caught in dust cloud:	Yes vs No*
		e	Worked in dust cloud:	Yes vs No*
	Injury	b	Sustained injury on 9/11:	Yes vs No*
	(Yes vs No*)	d	Injured on 9/11:	Yes vs No*
	Work Duration	b	>3 months	Days worked in any WTC site: level 1:1-7;
	(Long		vs otherwise (sum of levels 1–3) *	level 2: 8–30;level 3: 31–90;level 4: >90
	vs	e	≥ the top quartile (1353 hours	or 1.89 months) vs otherwise *
	Otherwise*)	g	≥ the median (total hours worked	608 hours or 0.84 months) vs otherwise *
		i	>5.5 months (level 5)	Time at site: level 1: ≤2 weeks;
			vs	level 2: up to 1.5 months;
			otherwise (sum of levels 1–4)*	level 3: up to 3 months;
				level 4: up to 5.5 months;
				level 5: >5.5 months
psychosocial	Lost Someone	b	Lost someone (sum of levels 1–4)	Loss/death of other on 9/11:
exposure	(Yes vs No*)		vs	level 1: Spouse; level 2: Other family member;
			None *	level 3: Coworker; level 4: Acquaintance;
				level 5 : None*
		g	Lost someone on 9/11:	Yes vs No*
	Witnessed Horror	b	Witnessed traumatic or	horrific event on 9/11: Yes vs No*
	(Yes vs No*)	c	Witnessed horror on 9/11:	Yes vs No*
		g	Exposed to human remains:	Yes vs No*

**Note**: Dichotomized exposure indicators were derived from exposure classifications used in the original studies. * indicates the reference group. Details of studies (a–j) were shown in Table 1.

It is also worth noting that large variations existed with regard to how WTC exposures were classified among the original studies (Table 2). For instance, studies from the FDNY (a, h, and j) and Registry (b) both used a 4-level exposure variable to define “arrival time”, while a 2-level “arrival time” was used in the two WTC-HP studies (g and i). Furthermore, large differences were also found in the specific cut-off points and the overall time durations covered in these studies (Table 2). To reconcile the discrepancies in the original exposure characterizations, we derived a dichotomized variable for each exposure using the lower or no exposure as the reference group in calculating probable PTSD risks (Table 2, e.g. late arrival, short work duration, and absent of exposure). Among these exposure types, “arrival time” and “work duration” were unique to responders, while others (“dust cloud”, “injury”, “lost someone”, and “witnessed horror”) were shared by both responders and civilians.

### Assessment of PTSD

Because all these PTSD assessments were based on self-report and not clinical diagnostics, the PTSD referred to in this study is probable PTSD. All but one [1] of the studies included in this meta-analysis administered the 17-item PTSD Checklist-Civilian (PCL) with the WTC as the focal event. The PCL assesses PTSD symptom severity for the last 30 days on a scale of 1 = not at all to 5 = extremely. Probable PTSD was determined using a cut-off point for the total severity score, or by severity scores congruent with the symptom criteria of Diagnostic and Statistical Manual of Mental Disorders, 4^th^ edition (DSM-IV,[30]), namely, at least 1 B item (question 1–5, for intrusion symptoms rated moderate-severe), plus 3 C items (questions 6–12, for avoidance/numbing symptoms), and plus 2 D items (questions 13–17, for hyperarousal symptoms). The one exception was a FDNY study [1] in which a modified PCL was administered, which has been validated for use in firefighters and detailed elsewhere [1] [6] [29]. Briefly, 14 of the 17 standard PCL symptoms were included in the modified version, the answers were rated on a binary scale (i.e. did or did not experience each symptom), and a cutoff of ≥9 (out of 14) was determined to be equivalent to a cutoff score of 44 in conjunction with the DSM-IV symptom criteria. As shown in Table 1, among the 10 studies, probable PTSD was operationalized by a cut-off score of 44 or 50 with or without the presence of DSM-IV criterion symptoms, and the PTSD prevalence ranged from 5.4% to 29.2% (Table 1).

### Effect size analysis

Based on the exposure classification, a total of 37 ORs (each OR was assigned to a unique internal identification in the analysis, Table S1) were available from the 10 studies for the final meta-analysis. We first calculated the overall summary of PTSD risk regardless of the specific WTC exposure type. The result showed that the overall PTSD risk was 2.05 [1.82, 2.32] with substantial heterogeneity (p-value <0.001 for the Cochran Q test). The I^2^ statistics (0.97 [0.96, 0.98]) was close to 100% and H^2^>5 (i.e. 31 [23, 59]). The between-study variance (τ^2^) was 0.14 (SE = 0.04). The heterogeneity was partly due to the influence of potential moderators (p-value <0.01, mixed-effects models), such as cohort type, WTC program, PTSD measure, and lapse time since 9/11, respectively. Most notably, responders had a significantly lower estimated OR than civilians (p<0.001), and the Registry had a higher OR than the FDNY (p<0.01).

**Figure 2:**
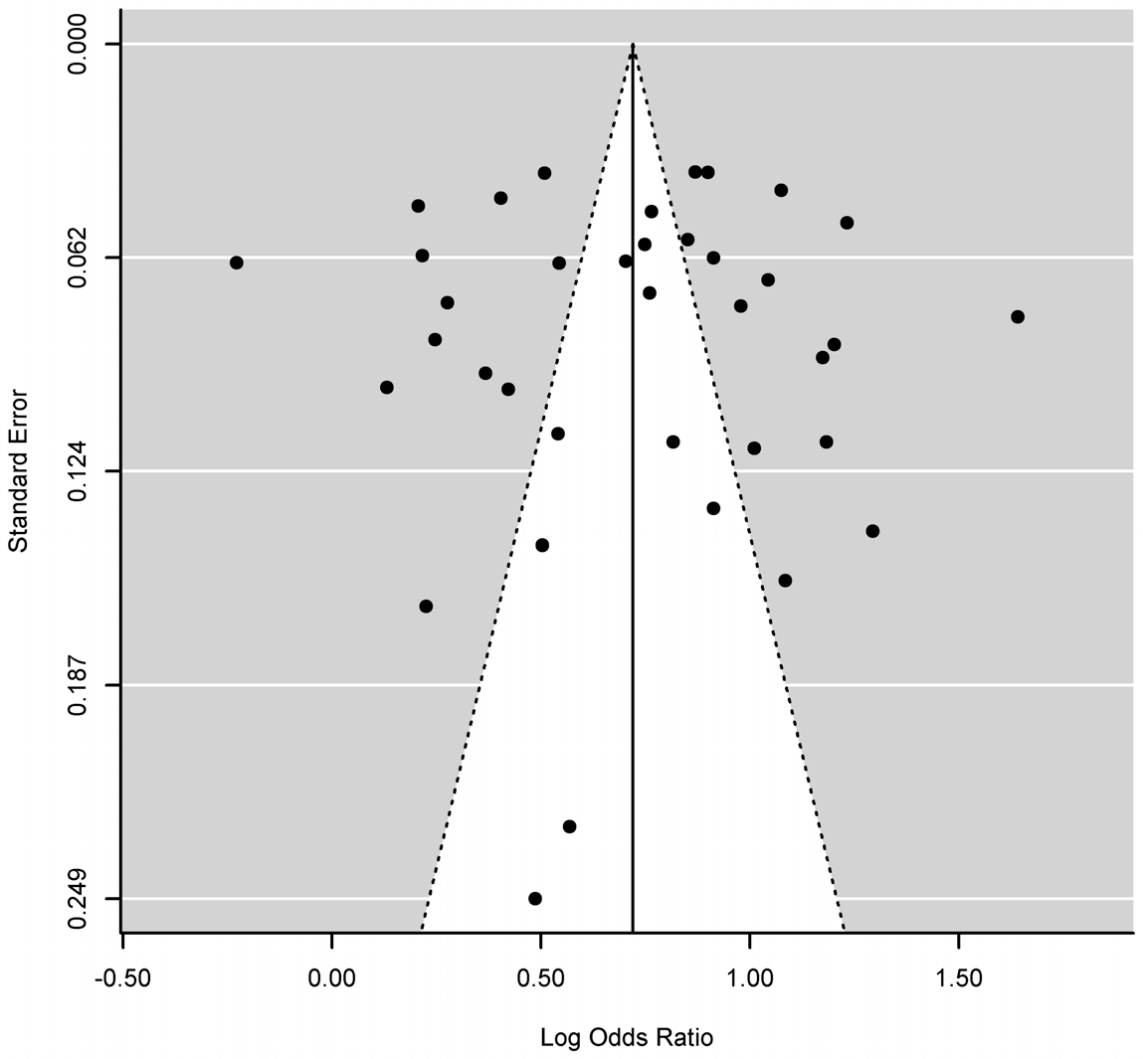
Funnel plot of the log odds ratios (ORs) of probable PTSD risks associated with WTC-related exposure for the meta-analysis of the ten studies included in the meta-analysis. Note: The points correspond to the 37 individual ORs. The funnel shape indicates the expected 95% confidence intervals around the summary estimate (vertical line). Little evidence of publication bias was found based on the symmetry of the funnel plot, which was also confirmed by both the Begg's test (p-value = 0.89) and Egger's test (p-value  =  0.93).

We found little evidence of publication bias from visually examining the symmetry of the funnel plot (Figure 2), which was also confirmed by both the Begg's test (p-value = 0.89) and Egger's test (p-value  =  0.93). The sensitivity analysis showed no substantial changes in the estimated summary ORs, which ranged from 2.0 [1.78–2.25] to 2.11 [1.89–2.37] with the values of τ^2^ ranging from 0.11 to 0.12.

**Figure 3:**
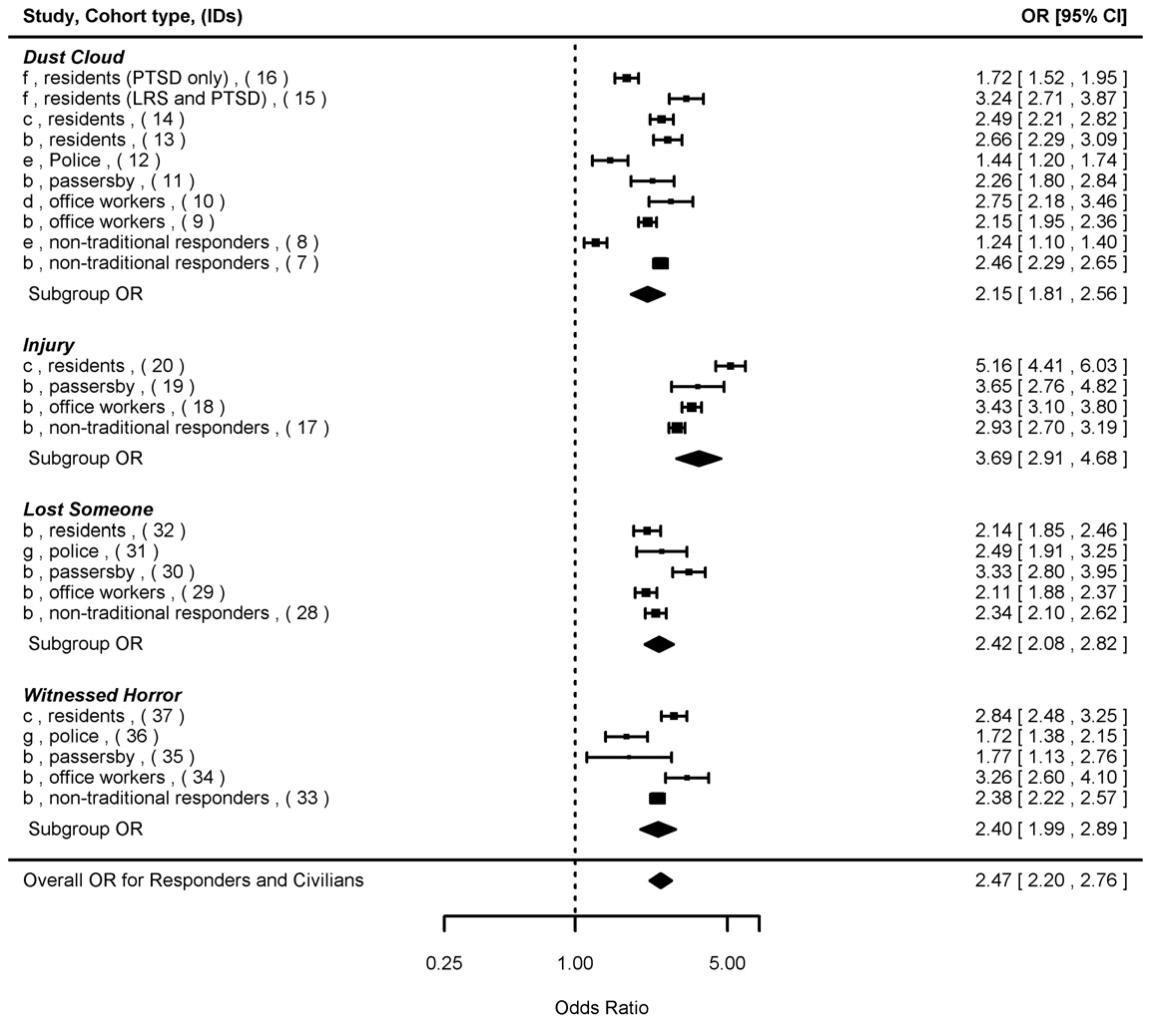
Forest plot of odds ratios (ORs and 95% confidence intervals) of probable PTSD risks associated with four specific WTC exposure types common between the responders and civilians. Note: Individual ORs from the original studies, summary ORs for the exposure subgroups, and the overall OR were presented. Details of the studies (a–j) and cohort types were shown in Table 1. IDs (1–37) corresponded to individual ORs in Table S1.

**Figure 4:**
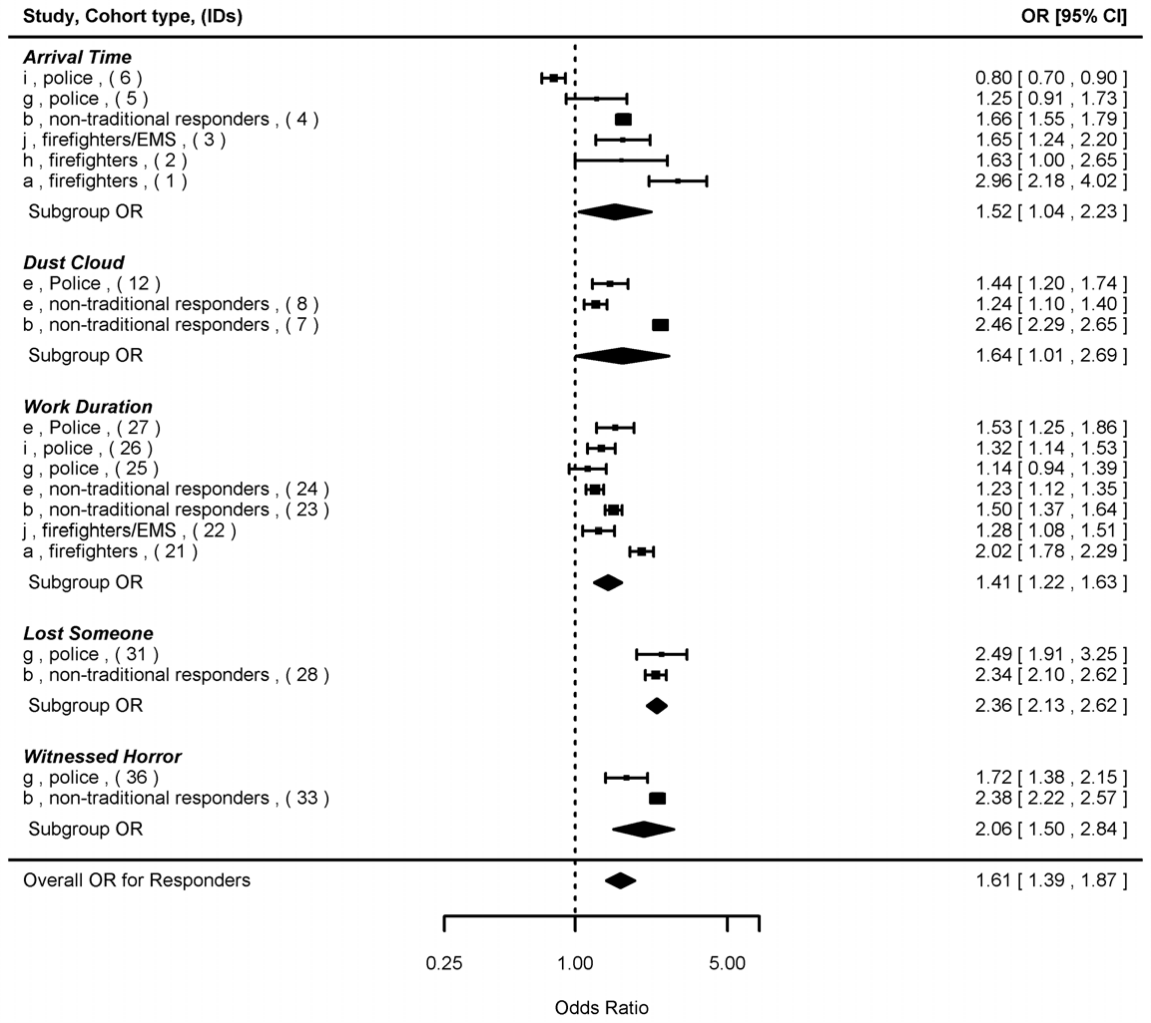
Forest plot of odds ratios (ORs and 95% confidence intervals) of probable PTSD risks associated with five specific WTC exposure types common among the responders. Note: Individual ORs from the original studies, summary ORs for the exposure subgroups, and the overall OR were presented. Details of the studies (a–j) and cohort types were shown in Table 1. IDs (1–37) corresponded to individual ORs in Table S1.

**Figure 5:**
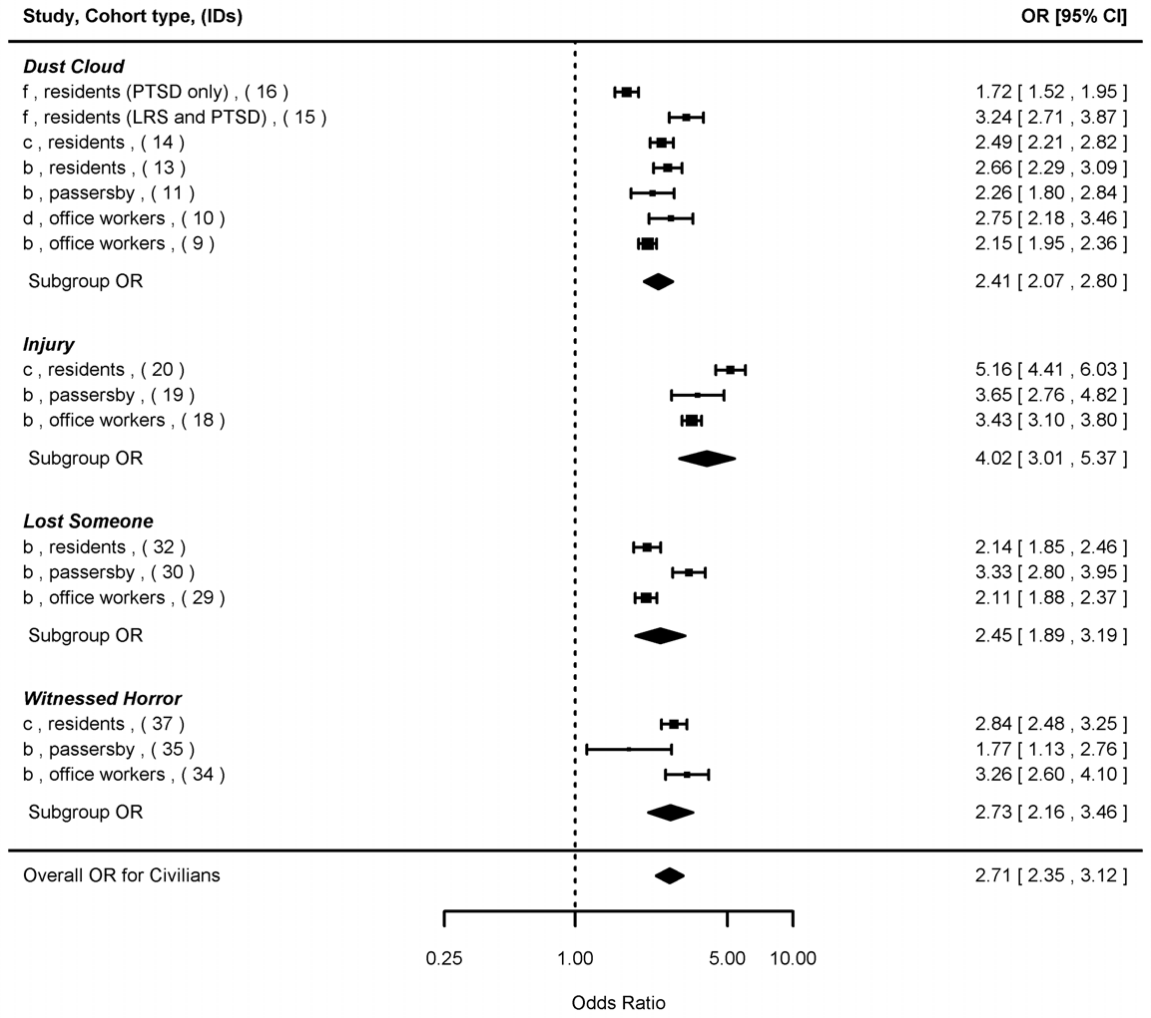
Forest plot of odds ratios (ORs and 95% confidence intervals) of probable PTSD risks associated with four specific WTC exposure types common among the civilians. Note: Individual ORs from the original studies, summary ORs for the exposure subgroups, and the overall OR were presented. Details of the studies (a–j) and cohort types were shown in Table 1. IDs (1–37) corresponded to individual ORs in Table S1.

We next summarized the ORs with respect to the exposure sub-groupings that were common to both responders and civilians (Figure 3), and also stratified by responders (Figure 4) and civilians (Figure 5), respectively. When both responder and civilian data were combined based on the four common exposure types (Figure 3), the overall summary OR was 2.47 [2.20, 2.76] (n = 24), with the highest OR found for exposure to “injury” (3.69 [2.91, 4.68]), and the lowest for “dust cloud” exposure (2.15 [1.81, 2.56]). The mixed-effects model showed significant influence (p<0.05) of cohort type (responders < civilians, p<0.001), WTC program (WTC-HP < Registry, p<0.001), and PTSD measure (PCL cutoff score of 50<44, p<0.001), but not from lapse time (p = 0.14).

For responders (Figure 4), the individual ORs associated with psychosocial exposure types ranged from 1.72 to 2.49 and all of the 95% CIs were above one, while ORs associated with physical exposure types ranged from 0.80 to 2.96 and some 95% CIs contained one. The summary OR among police and firefighters was 1.53 [1.25, 1.88] (n = 13), lower than that found among the non-traditional responders (1.88 [1.50, 2.34], n = 8). Among civilians (Figures 5), all the individual ORs, regardless of physical or psychosocial exposure types, were statistically significant. The highest summary ORs for civilians were seen in the “injury” exposure category (4.02 [3.01, 5.37]) followed by “witnessed horror” (2.73 [2.16, 3.46]). The ORs for “dust cloud” (2.41 [2.07, 2.80]) and “lost someone” (2.45 [1.89, 3.19]) exposures were similar. When the overall summary ORs were compared by cohort types, stronger associations were found for civilians (2.71 [2.35, 3.12], n = 16) compared to responders (1.66 [1.42, 1.94], n = 21).

Results from the additional analysis on a subset that consisted of 25 ORs (Table S1) showed similar associations seen in the full data set. WTC exposure were significantly associated with probable PTSD with an overall OR of 2.17 [1.88, 2.51] and ranging from 2.56 [2.30, 2.84] for both responders and civilians combined (Figure S1), 1.67 [1.37, 2.03] among the responders (Figure S2), and to 2.78 [2.36, 3.28] among the civilians (Figure S3).

## Discussion

This meta-analysis used pooled data from WTC-related studies to evaluate and compare the probable PTSD risk associated with specific exposures among adults in the greater New York area. We found that the overall summary OR, the summary ORs by cohort types, and summary ORs for specific types of exposures from the ten studies reviewed herein were all statistically significant. This analysis confirms results from the existing body of evidence showing strong associations between a variety of WTC exposures and risk of probable PTSD for both responders and civilians.

There are several challenges to be considered in the research on associations between the WTC exposure and PTSD. Both variations in the nature of the exposure (e.g. specific type, duration, and severity of trauma exposure) and in the status of the affected individuals (e.g. age when exposure occurred, sex, education, occupation, psychiatric and physical comorbidity, coping mechanisms and capability, etc.) may influence the PTSD outcome [5] [7] [9] [11] [13] [31]. In terms of characterizing the WTC exposure, given the magnitude of the impact of 9/11 and the diverse population affected by the event, it is not surprising that we found diverse exposure types across the relevant studies. However, only a handful of specific exposure types overlapped among these studies. It is also worth noting that there were large variations among the original exposure classifications in terms of exposure severity and specificity. In general, the exposure classifications were more consistent in the responder research, particularly within individual health programs, than civilian studies. For example, “arrival time” and “work duration” were ascertained in research on police, firefighters, and non-traditional responders, though different cutoffs were used in determining duration of the exposure among the three responder types. As a result, the severity of the WTC exposure and the consequent PTSD health outcome also varied. These discrepancies undoubtedly contributed to the large heterogeneity seen among the ORs.

In general, the associations between exposure and probable PTSD were weaker in responders than civilians, and this difference was more pronounced for physical compared to psychosocial exposure types. On one hand, the responders faced unprecedented, treacherous working conditions at the site and were potentially under more intense physical and psychological stress. Thus, one could hypothesize that the risk of having PTSD might be higher in responders than civilian populations. On the other hand, the police and firefighters were professionally trained to work under dangerous situations, and thus had greater experience coping with disasters than civilians. Indeed, we found an overall weaker exposure-PTSD association among police and firefighters compared to the non-traditional responders (OR of 1.53 vs. 1.88), consistent with results from other studies showing traditional responders with training had lower rates of probable PTSD than non-traditional responders without prior training [32]. In this study, the benefit of training and experience was certainly reflected in the lower OR ranges seen for the physical exposure types (“dust cloud” and “injury”) among the responders (1.24–2.46 and 2.93, respectively) compared to the civilians (1.72–3.24 and 3.43–5.16, respectively). It was also reflected in the overall low and non-significant ORs in the work-related exposure among responders, with ORs ranging from 0.80 to 2.96 and from 1.14 to 2.02 for the “arrival time” and “work duration”, respectively. However, it is likely that these two physical exposure types were less sensitive in predicting PTSD compared to exposures with direct link to mental stress. Indeed, when psychosocial exposure types were considered, limited differences in ORs were found between responders and civilians. For the “lost someone” and “witnessed horror” exposure types, the associations ranged from ORs of 2.34–2.49 and 1.72–2.38 among responders, respectively, to 2.11–3.33 and 1.77–3.26, respectively, among civilians. Police and firefighters reporting these losses often sustained multiple losses of close colleagues, with entire work units massively affected. Thus, future studies of loss need to distinguish between the nature and number of losses sustained during this horrendous event.

Apart from heterogeneity in the classification of WTC exposure, other factors could also influence the relatively weaker overall exposure-PTSD associations found in responders compared to civilians. In this study, a dominant proportion of traditional responders and a majority of the non-traditional responders were males and whites, while the sociodemographic profiles of the civilian groups were more diverse. Studies have shown a general elevated prevalence in PTSD and other anxiety and mood symptoms among females compared to males and/or among Hispanic ethnicity [2] [4] [17] [31] [33] [34]. Other studies have argued that concerns of repercussions could also lead to underreporting of mental health symptoms among police [35].

This paper sheds new light on the associations of WTC exposure to probable PTSD by providing quantitative estimates of the associations as indicated by ORs from the existing 9/11-related research accumulated over more than a decade. We identified three exposure types (i.e. injury, loss of life, and witnessed horror) out of six to be associated with greater PTSD risks, suggesting they should be included in emergency preparation, evaluation, and treatment programs of future disasters for both responders and civilians. Our results also showed differences in the PTSD risks were attributable to diverse exposure classifications and cohort types, as well as other moderator such as data sources, PTSD assessment instrument/criteria, and lapse time since 9/11.

Our results must also be considered in relation to study limitations. First, our meta-analysis was constrained by the availability of only 10 studies that met our selection criteria. While the summary effect size was based on 15-37 individual ORs, we also had a few sub-exposure-group analyses that were based on only 2 data points. Thus caution must be taken in drawing inferences for the subgroup summaries based on these small numbers. Second, we only estimated crude ORs for the WTC exposure variables without adjusting for other factors, such as age, sex, socioeconomic status, and co-morbidity, which have been shown to affect PTSD outcomes [31] [32] [36] [37]. While changes in the summary ORs may be limited, as the significant associations were still present after adjusting for relevant covariates in most studies included in this meta-analysis, influences of these potential moderators and others that were not considered in the current study deserve further attention by ongoing WTC studies. Third, there was a modest overlap in the WTC-HP and Registry samples, and potentially among studies from the same data source. We attempted to minimize the impact of the shared sample by careful selection of studies and sub-analyses, which produced findings that were comparable to the overall results of the meta-analysis. Finally, the studies reviewed here relied on a self-reported PTSD symptom scale rather than diagnostic interviews, and volunteer samples. We note that the Stony Brook site of the WTC-HP found comparable prevalence rates for PCL>50 and diagnostic assessment of PTSD, and good sensitivity and specificity [38] . While the samples are volunteers, they are large and diverse, and thus the findings are based on broadly obtained symptom and exposure data assessed at varied time points since 9/11. Nevertheless, memory for traumatic event is not fixed and caution must be taken in assessing the accuracy of the recall for traumatic events and the subsequent relationships between stressors and PTSD [39].

### Conclusions

Our meta-analysis of ten studies demonstrates significant positive associations between the WTC exposure and probable PTSD across six common exposure categories and the two major cohort types examined. The strength of the associations appeared to be lower among responders as compared to civilians. This difference was more pronounced for physical compared to psychosocial exposure types, suggesting while professional experience and training played an important role in predicting PTSD, other factors may also influence the risk of PTSD, such as heterogeneity of the exposure classification, data source, PTSD cut-point, lapse time since 9/11, as well as differences in sociodemographic profiles.

We also found that injury, lost someone, and witnessed horror were the three strongest predictors of probable PTSD among those affected by the 9/11 terrorist attack, regardless of cohort types. Given the consistency of this finding across populations, patients seeking treatment for 9/11 associated health problems should be asked about experiences of injuries, losses, and witnessing of horror as part of their assessment and providers should be aware of the long-term effect of these exposures on PTSD so that appropriate interventions can be offered. Emergency preparations for future disasters should anticipate these three specific exposures as potential risk factors of persistent PTSD symptoms in both responders and civilians.

Finally, the search resulted in surprisingly few studies met our criteria for this meta-analysis. This scarcity of data highlights the challenge of conducting post-disaster health assessment (sometimes based on quickly developed questionnaires that are prone to lack of compatibility among studies) and the challenges inherent in subsequent services and research efforts to understand both short- and long-term health effects. Thus, psychosocial surveillance techniques such as questionnaires that are able to distinguish between the nature and severity of exposure types should be developed in advance to improve the consensus in the assessment of specific exposure and health endpoints. We also recommend that future studies of WTC responders and civilians provide more specific information on exposure and mental health outcomes so that meta-analyses of long-term effects can encompass a broader array of studies in order to develop and modify existing response and recovery plans, and to prepare and mitigate for future disasters.

## Supporting Information

Figure S1 **Forest plot of odds ratios (and 95% confidence intervals) of self-reported PTSD risks associated with four specific WTC exposure types common between the responders and civilians.** The effect size analysis was based on a subset of data, and showed similar results to those found using the full data set. Notes: Individual ORs from the original studies, summary ORs for the exposure subgroups, and the overall OR were presented. Details of the study numbers (a–j) and cohort types were defined in Table 1. IDs corresponded to a unique internal identification of ORs used in the analysis as shown in Table S1.
(TIFF)

Figure S2 **Forest plot of odds ratios (and 95% confidence intervals) of self-reported PTSD risks associated with five specific WTC exposure types among the responders.** The effect size analysis was based on a subset of data, and showed similar results to those found using the full data set. Notes: Individual ORs from the original studies, summary ORs for the exposure subgroups, and the overall OR were presented. Details of the study numbers (a–j) and cohort types were defined in Table 1. IDs corresponded to a unique internal identification of ORs used in the analysis as shown in Table S1.
(TIFF)

Figure S3 **Forest plot of odds ratios (and 95% confidence intervals) of self-reported PTSD risks associated with four specific WTC exposure types among the civilians.** The effect size analysis was based on a subset of data, and showed similar results to those found using the full data set. Notes: Individual ORs from the original studies, summary ORs for the exposure subgroups, and the overall OR were presented. Details of the study numbers (a–j) and cohort types were defined in Table 1. IDs corresponded to a unique internal identification of ORs used in the analysis as shown in Table S1.
(TIFF)

Table S1 **Detailed data (n = 37) extracted from the ten original studies included in the meta-analysis, where sample numbers with and without self-reported PTSD (P+/P-) among those with high WTC exposure vs reference levels (low or no) exposure were shown.** A subset (*, n = 25) of data was used to further investigate the impact of shared sample issues. Similar results were found for effect size analyses based on both data sets.
(DOCX)

Checklist S1 **PRISMA checklist.**
(DOC)
