# Supplementary material for: World Trade Center Disaster Exposure-Related Probable Posttraumatic Stress Disorder among Responders and Civilians: A Meta-Analysis
Source: PLoS One. 2014 Jul 21;9(7):e101491. doi: 10.1371/journal.pone.0101491 (PMC4105417; doi:10.1371/journal.pone.0101491)
Supplement: Table S1 — Detailed data (n = 37) extracted from the ten original studies included in the meta-analysis, where sample numbers with and without self-reported PTSD (P+/P-) among those with high WTC exposure vs reference levels (low or no) exposure were shown. A subset (*, n = 25) of data was used to further investigate the impact of shared sample issues. Similar results were found for effect size analyses based on both data sets. (DOCX) [file pone.0101491.s004.docx]

**Table S1.** Detailed data (n=37) extracted from the ten original studies included in the meta-analysis, where sample numbers with and without self-reported PTSD (P+/P-) among those with high WTC exposure vs reference levels (low or no) exposure were shown. A subset (*, n=25) of data was used to further investigate the impact of shared sample issues. Similar results were found for effect size analyses based on both data sets.

| **Study** | | **Sample Type** | **P+** | **P-** | **Reference P+** | **Reference P-** | **Exposure Type** | **ID** |
| --- | --- | --- | --- | --- | --- | --- | --- | --- |
| a | Berninger et al., 2010 | Firefighters | 1409 | 7876 | 45 | 744 | arrival time | 1 |
| h | Soo et al., 2011 | firefighters | 315 | 3669 | 18 | 341 | arrival time | 2 |
| j | Webber et al., 2011* | firefighters/EMS | 699 | 8968 | 53 | 1125 | arrival time | 3* |
| b | Brackbill et al., 2009* | non-traditional responders | 1479 | 4258 | 2379 | 11394 | arrival time | 4* |
| g | Pietrzak et al., 2012 | police | 379 | 5507 | 44 | 801 | arrival time | 5 |
| i | Stellman et al., 2008* | police | 627 | 5519 | 498 | 3488 | arrival time | 6* |
| b | Brackbill et al., 2009* | non-traditional responders | 1793 | 4405 | 1887 | 11402 | dust cloud | 7* |
| e | Luft et al., 2012 | non-traditional responders | 415 | 1152 | 2422 | 8345 | dust cloud | 8 |
| b | Brackbill et al., 2009* | office workers | 2049 | 6864 | 650 | 4678 | dust cloud | 9* |
| d | DiGrande et al., 2011 | office workers | 387 | 1598 | 102 | 1157 | dust cloud | 10 |
| b | Brackbill et al., 2009* | passersby | 325 | 827 | 128 | 737 | dust cloud | 11* |
| e | Luft et al., 2012* | Police | 183 | 2276 | 319 | 5730 | dust cloud | 12* |
| b | Brackbill et al., 2009 | residents | 563 | 1885 | 316 | 2813 | dust cloud | 13 |
| c | DiGrande et al., 2008* | residents | 974 | 4687 | 409 | 4906 | dust cloud | 14* |
| f | Nair et al., 2012 | residents (LRS and PTSD) | 792 | 9587 | 147 | 5758 | dust cloud | 15 |
| f | Nair et al., 2012 | residents (PTSD only) | 1027 | 9352 | 354 | 5551 | dust cloud | 16 |
| b | Brackbill et al., 2009* | non-traditional responders | 1125 | 1957 | 2825 | 14401 | injury | 17* |
| b | Brackbill et al., 2009* | office workers | 774 | 1185 | 2040 | 10710 | injury | 18* |
| b | Brackbill et al., 2009* | passersby | 114 | 125 | 370 | 1480 | injury | 19* |
| c | DiGrande et al., 2008* | residents | 304 | 497 | 1085 | 9151 | injury | 20* |
| a | Berninger et al., 2010 | firefighters | 665 | 2604 | 530 | 4192 | work duration | 21 |
| j | Webber et al., 2011* | firefighters/EMS | 322 | 4088 | 261 | 4239 | work duration | 22* |
| b | Brackbill et al., 2009* | non-traditional responders | 898 | 2275 | 2695 | 10230 | work duration | 23* |
| e | Luft et al., 2012 | non-traditional responders | 851 | 2454 | 1986 | 7042 | work duration | 24 |
| g | Pietrzak et al., 2012 | police | 232 | 3257 | 197 | 3154 | work duration | 25 |
| i | Stellman et al., 2008* | police | 265 | 1726 | 838 | 7194 | work duration | 26* |
| e | Luft et al., 2012 | Police | 153 | 1787 | 349 | 6219 | work duration | 27 |
| b | Brackbill et al., 2009* | non-traditional responders | 2831 | 8945 | 407 | 3013 | lost someone | 28* |
| b | Brackbill et al., 2009* | office workers | 2044 | 7159 | 407 | 3013 | lost someone | 29* |
| b | Brackbill et al., 2009* | passersby | 297 | 661 | 407 | 3013 | lost someone | 30* |
| g | Pietrzak et al., 2012* | police | 375 | 4556 | 67 | 2028 | lost someone | 31* |
| b | Brackbill et al., 2009* | residents | 520 | 1801 | 407 | 3013 | lost someone | 32* |
| b | Brackbill et al., 2009* | non-traditional responders | 2639 | 7472 | 1306 | 8818 | witnessed horror | 33* |
| b | Brackbill et al., 2009* | office workers | 2726 | 10769 | 83 | 1070 | witnessed horror | 34* |
| b | Brackbill et al., 2009* | passersby | 460 | 1465 | 24 | 135 | witnessed horror | 35* |
| g | Pietrzak et al., 2012* | police | 319 | 4026 | 110 | 2385 | witnessed horror | 36* |
| c | DiGrande et al., 2008* | residents | 1096 | 5468 | 295 | 4178 | witnessed horror | 37* |
